# Vascular Dementia and Underlying Sex Differences

**DOI:** 10.3389/fnagi.2021.720715

**Published:** 2021-09-08

**Authors:** Firoz Akhter, Alicia Persaud, Younis Zaokari, Zhen Zhao, Donghui Zhu

**Affiliations:** ^1^Department of Biomedical Engineering, Stony Brook University, Stony Brook, NY, United States; ^2^Department of Physiology and Neuroscience, Zilkha Neurogenetic Institute, Keck School of Medicine, University of Southern California, Los Angeles, CA, United States; ^3^Neuroscience Graduate Program, Renaissance School of Medicine, Stony Brook University, Stony Brook, NY, United States

**Keywords:** vascular dementia, Alzheimer’s disease, sex, gender, multi-infarct dementia

## Abstract

Vascular dementia (VaD) is the second most common form of dementia after Alzheimer’s disease (AD); where Alzheimer’s accounts for 60–70% of cases of dementia and VaD accounts for 20% of all dementia cases. VaD is defined as a reduced or lack of blood flow to the brain that causes dementia. VaD is also known occasionally as vascular contributions to cognitive impairment and dementia (VCID) or multi-infarct dementia (MID). VCID is the condition arising from stroke and other vascular brain injuries that cause significant changes to memory, thinking, and behavior, and VaD is the most severe stage while MID is produced by the synergistic effects caused by multiple mini strokes in the brain irrespective of specific location or volume. There are also subtle differences in the presentation of VaD in males and females, but they are often overlooked. Since 1672 when the first case of VaD was reported until now, sex and gender differences have had little to no research done when it comes to the umbrella term of dementia in general. This review summarizes the fundamentals of VaD followed by a focus on the differences between sex and gender when an individual is diagnosed. In addition, we provide critical evidence concerning sex and gender differences with a few of the main risk factors of VaD including pre-existing health conditions and family history, gene variants, aging, hormone fluctuations, and environmental risk factors. Additionally, the pharmaceutical treatments and possible mitigation of risk factors is explored.

## Introduction

Vascular dementia (VaD), a heterogeneous group of brain disorders is the next most common form of dementia following Alzheimer’s disease (AD) and accounts for at least 20% of dementia cases (Román, 2003; Korczyn et al., 2012). VaD is caused by a blocked or reduced blood flow to the brain which will deprive neurons of critical nutrients (Vijayan and Reddy, 2016). This deprivation eventually causes the neurons to die, and the brain tissue starts to shrink. Some common contributors to this kind of dementia include stroke, cardiovascular disease, diabetes, hyperlipidemia, and hypertension (Song et al., 2014).

Vascular cognitive impairment (VCI) is a term that encompasses a continuum of cognitive disorders with cerebrovascular pathology contribution, ranging from mild cognitive impairment to VaD (Nguyen et al., 2021). As a result, VCI and VaD constitute an intriguing junction of cardiovascular disease (CVD), and neurodegenerative disorders, like AD, are a growing topic of research in recent years. Even though VCI and VaD research has identified a variety of causes and explanations for disease development, many aspects remain unknown, particularly sex differences in VCI (e.g., epidemiology), which are lacking in comparison to those available for CVD and AD.

### History of Vascular Dementia (VaD)

The root meaning of the word dementia came from the Latin word demons which means “Without mind.” The first case of VaD was reported in 1672 by Thomas Willis in his book De Anima Brutorum (Román, 2003). After the recognizing of the differences between hemorrhage and ischemic stroke, Bayle discovered that apoplexy was the obstruction of the arteries with effusion of blood in such small amounts because of the failure of the blood influx to the brain (Engelhardt, 2017). Apoplexy is unconsciousness and/or incapacitation resulting from a cerebral hemorrhage or stroke. Later, apoplexy was considered the cause of brain congestion or cerebral hyperemia in most of the 18th and early 19th centuries. The year 1894 is considered to be the beginning of the modern history of VaD due to Otto Binswanger and Alois Alzheimer, who were the first to separate the forms of dementia from neurosyphilis and recognize a heterogeneous VaD (Yang et al., 2016). In 1910 Emile Kraepelin closely observed the concept of Otto Binswanger and Alois Alzheimer with a different description for VaD as arteriosclerotic dementia or cerebral arteriosclerosis (Román, 2003). For 70 years after Kraepelin’s description, the synonym for his description of VaD was called senile dementia. Hachinski et al. (1974) emphasized that vascular disease was responsible for dementia, via the occurrence of small or large cerebral infarcts; the term Multi Infract dementia (MID) was to be used synonymously with VaD. In 1995 a proposal was made to the National Institutes of Health that the broader term VaD could be changed to the new name Vascular Cognitive Impairment.

### Symptoms, Risk Factors, Characteristic Features, and Subtypes of VaD

**FIGURE 1:**
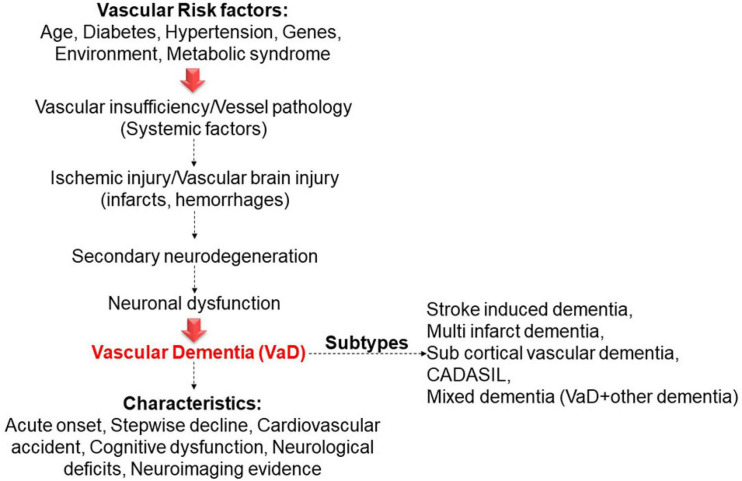
Subtypes of VaD that are associated with some of the vascular risk factors and their characterized features.

The symptoms of VaD differ depending on which part of the brain the vessels and blood flow are obstructed (Venkat et al., 2015). However, the common indications might appear as disorientation, difficulty thinking, understanding, inability to create new memories, agitation, or behavioral symptoms (Kalaria, 2016). Moreover, VaD symptoms may be most obvious when they happen soon after a major stroke, and they can gradually develop (Dichgans and Leys, 2017). Figure 1 shows some of the risk factors, characteristic features, and subtypes associated with VaD.

The risk factors help perpetuate the occurrence of the VaD. Strokes that block a brain artery usually cause a range of symptoms like cognitive decline, neurological deficits, and further severe cardiovascular incidents that may include vascular dementia (Shabir et al., 2018). When VaD begins, it can be classified into various subtypes that are typically related to the root cause of the VaD (Rockwood et al., 1999). Stroke, multi-infarct, and subcortical dementia refer to the vascular events leading to dementia (Kalaria and Erkinjuntti, 2006). CADASIL refers to a subtype of VaD, in which a specific gene variant must be present (Benisty et al., 2008). This variant whilst mixed with dementia causes a rare disease subtype that refers to the presence of multiple forms of dementia including the presence of vascular events (Benisty et al., 2008). In CADASIL, migraine with aura is more frequent in women and, stroke is more frequent in men before menopause (Gunda et al., 2012). After this age limit, the difference appears to vanish, however, men in the late stages of the disease may experience more cognitive impairment and cerebral atrophy.

Dementia is a consequence and a risk factor for stroke and VaD. Stroke is one of the primary causes of disability, and having a stroke doubles your risk of developing VaD (Vijayan and Reddy, 2016). Lower education, older age, diabetes mellitus, myocardial infarction, atrial fibrillation, epileptic seizures, sepsis, cardiac arrhythmias, congestive heart failure, global cerebral atrophy, and medial temporal lobe atrophy, and white matter changes have all been linked to an increased risk of dementia after a stroke (Pinkston et al., 2009). Post-stroke depression is another risk factor for VaD, which is more common in women than in men (Pendlebury and Rothwell, 2009). Female sex, medial temporal lobe atrophy, and a family history of dementia are all better predictors of pre-stroke dementia than post-stroke dementia (Podcasy and Epperson, 2016). Pre-stroke dementia could be a symptom or cause of a fundamental degenerative condition that makes vascular events more likely (Glader et al., 2003).

Multi-infarct dementia is a type of VaD, occurs when a series of minor strokes triggers a loss of brain function (Al-Qazzaz et al., 2014). A stroke, or brain infarct, occurs when the blood flow to any part of the brain is disrupted or blocked. Blood supplies oxygen to the brain, and brain tissue dies quickly without it. Memory and cognitive function loss can occur because of MID, as well as psychosocial issues. MID generally occurs in people aged 55 to 75 years and is more common in men than women (McKay and Counts, 2017). Medical conditions and lifestyle that increase the risk of MID include atrial fibrillation, rapid heartbeat, previous strokes, heart failure, cognitive decline before a stroke, high blood pressure, diabetes, atherosclerosis, or hardening of the arteries, smoking, alcohol, a low level of education, a poor diet, little to no physical activity (Lechner and Bertha, 1991).

Subcortical VaD is the subtype that has attracted the most interest among all the potential VaD subtypes (Wallin et al., 2003). Lacunar infarcts and ischemic white-matter lesions with demyelination and axon loss are the most common brain lesions in this kind of VaD. The primary cause is an injury to the penetrating arteries vessel walls (Kalaria, 2016). Multiple vascular problems, such as arterial hypertension, diabetes, and ischemic heart disease, are common in patients with subcortical VaD. Impairment goal formulation, starting, planning, and organizing are among the clinical signs (O’Brien and Thomas, 2015). Cerebrovascular white matter lesions are associated with stroke, cognitive decline, dementia, and death in a meta-analysis of longitudinal studies (Debette and Markus, 2010). These lesions correlate with advancing age, female gender, and vascular risk factors, including hypertension, diabetes mellitus, smoking, and lower income (Tomimoto, 2011). Another study was unable to find significant differences in the role of sex in subcortical VaD cases (Choi et al., 2020).

Cognitive impairment caused by numerous central nervous system (CNS) disorders are referred to as mixed dementia. A combination of AD pathologies such as amyloid deposits and tau tangles—and vascular impairment, such as numerous microbleeds or infarcts, is the most common cause of this disease (Jellinger, 2004). Rendering to vascular theory, chronic diseases (hypertension, diabetes mellitus, cardiac disease, dyslipidemia, and obesity) and the sedentary lifestyle produce numerous vascular changes, which generate vascular atrophy of the vascular terminations, as well as a reduction in the number of terminal blood vessels (Zekry et al., 2002). These alterations affect the cerebral microvasculature and diminish the cerebral blood flow, which is mainly observed in untreated hypertensive patients and those treated sporadically or inadequately (Hanyu, 2012). It is generally accepted that vascular dementia and mixed dementias occur more frequently in males, with rates of 31 vs. 25% in females (Podcasy and Epperson, 2016).

### Diagnosis, and Current Treatment

The diagnostic tools for VaD vary on the diagnosis of the patients. A person suspected of having VaD will generally have a brain scan to look for any changes that have taken place in the brain. A scan such as Computerized tomography (CT) or magnetic resonance imaging (MRI) is also common to visualize brain function and may rule out a blockage or build-up of fluid inside the brain (van Straaten et al., 2004). Carotid ultrasound is used to determine whether the carotid artery shows any signs of narrowing because of plaque deposits or structural issues causing reduced blood flow to the brain (Malojcic et al., 2017). Neurological exams for reflexes, muscle tone and strength, are used as well as qualitative exams on comparing strength on one side of the body with the other side, assessing ability to get up from a chair and walk across the room, the intensity of touch, sight, coordination, and balance. Neuropsychological tests are also used to examine the ability to speak, write and understand language, work with numbers, learn and remember information, develop a plan of attack/solve a problem, and respond effectively to hypothetical situations (Pimentel, 2009).

In terms of treatments, the medical field lacks actual drugs that are fully approved by the FDA. However, there are clinical trials for drugs that treat AD symptoms which can also be offered to patients with VaD as well (Huang et al., 2020). With regards to anti-dementia therapeutics, there are no specific drugs approved for VaD treatment. The cholinesterase inhibitors, and the NMDA (the N-methyl-D-aspartate receptor) antagonist, are the only medications currently licensed for AD treatment, have been found to show some cognitive improvements in mild to moderately advanced VaD (Kandiah et al., 2017; Yaowaluk et al., 2019; Jian et al., 2020). Memantine belongs to the aminoadamantane chemical class and is structurally like amantadine, an anti-Parkinson and antiviral drug. Memantine has been tested in a study that included 815 subjects with mild to moderately advanced VaD (Baskys and Hou, 2007). Treatment with 20 mg/day dose or placebo lasted 28 weeks. Data analysis showed a significant improvement in cognitive function, measured as ADAS-cog (Alzheimer’s Disease Assessment Scale-cognitive subscale), from baseline, over placebo. In a recent study (Arrigo et al., 1990), 56 patients with dementia and cerebrovascular illness were randomized to receive either acetyl-L-carnitine (ALC) 1.5 g/day or placebo for 28 weeks in a multicenter, double-blind, placebo-controlled clinical trial. The individuals’ scores on the Montreal Cognitive Assessment improved dramatically after taking ALC, particularly in the attention and language subitems. However, it’s difficult to believe that the study was solely focused on VaD because the patients were already on donepezil, implying that a significant proportion of them may have been affected by AD or mixed dementia (Pennisi et al., 2020). A small but statistically significant improvement was found on the NOSGER (Nurses Observational Scale for Geriatric Patients) disturbing behavior scale. Additionally, there have been attempts to use similar medicines that treat underlying causes of VaD. For example, in a small study hypertension medicines had a positive correlation with VaD risk reduction compared to subjects with similar risk factors but were without the antihypertensive (Iulita and Girouard, 2017). These include tablets to reduce blood pressure, prevent blood clots and lower cholesterol. Sometimes if treated early blood vessels can repair and slow the rate at which VaD progresses and might prevent further development of symptoms (Lee, 2011). A 5-year follow-up study on a community sample of 1617 African Americans demonstrated that the use of medications that mediate vascular risk factors (antihypertensive drugs, anti-hyperlipidemic, and antidiabetic drugs) reduced the risk of incident dementia by 40% (Kennelly et al., 2009). Lastly, some research also suggests that after a big vascular event like a stroke, preventative measures are vital for mitigating the risk of developing VaD (Vijayan and Reddy, 2016; Hachinski et al., 2019).

## Clinical Presentation of VaD

Since 2011, published studies are, not only in VaD but in some dementias and other cognitive disorders as well. However, a critical review of the more recent trials and the effects of sex/gender on VaD is still lacking. The purpose of this clinical presentation is to provide an update on the existing studies dealing with sex and gender in VaD to obtain a clear and comprehensive overview of all clinical benefits, potential effects of sex/gender, limitations, and main outcomes in the management of these patients.

### Data Source and Selection

A Medline (PubMed)-based literature review was performed by using the following search terms, in different combinations: “Vascular dementia,” “Vascular cognitive impairment,” “Vascular cognitive impairment and dementia,” “Arteriosclerotic dementia,” “multi-infarct dementia,” “vascular contributions to cognitive impairment and dementia,” “sex,” and “gender.” The studies had to include individuals with a clinical diagnosis of any type of Vascular dementia in relation to sex or gender and severity according to the internationally accepted guidelines or diagnostic criteria. Duplicated entries, studies on physiological brain aging or diseases different from VaD, VCI, or VCID, works on animals or cell cultures, studies not reporting the statistical analysis, non-English written papers, publications that are not research studies (i.e., abstracts, letters, commentaries, editorials, reviews, etc.), conference, meeting proceedings, or any other paper not published in international peer-reviewed journals, study protocols, personal communications, or unpublished data, as well as any other study that did not fit with the scope of this review were excluded. Articles listed in the references were also reviewed in search of more data.

### Results

**FIGURE 2:**
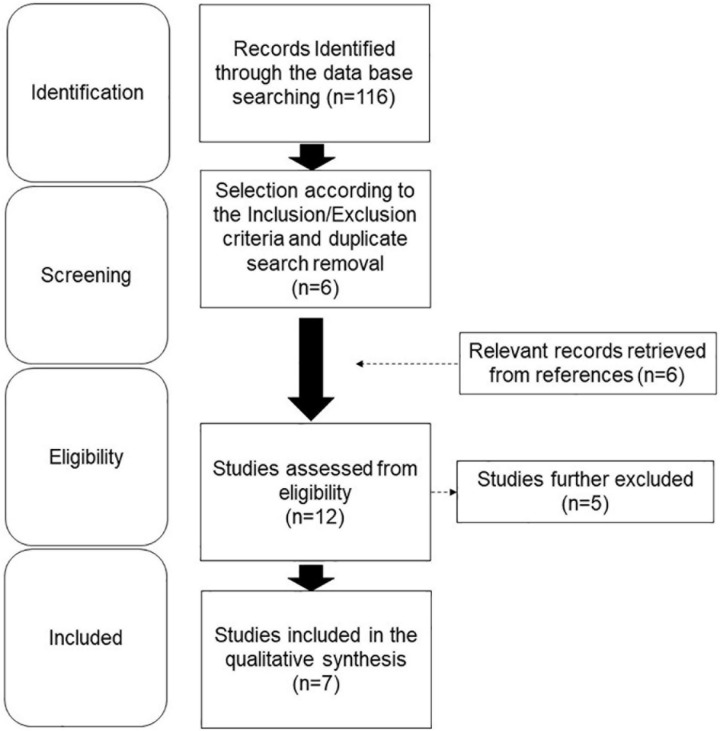
Flow diagram showing the search strategy, the number of records identified, and the number of included/excluded studies.

A total of 113 results were originally retrieved and screened. Of these, six publications were selected according to the inclusion and exclusion criteria. The examination of the references detected other five studies, whose analysis identified one additional paper fitting the purpose of this review. Therefore, a total of seven papers were eventually included in the qualitative synthesis (Figure 2), and the main findings are summarized in Table 1 (Vinciguerra et al., 2020).

**TABLE 1:** Studies on vascular dementia (VaD) in relation with sex or gender.

**Sex**	**Age in years (Average age)**	**Diagnostic criteria**	**Education and marital status**	**Medical risk factors**	**Subjects**	**Key findings**	**Adjusted variables**	**References**
Male	≥55.0 (80.3)	MMSE, GMS-B, AGECAT, HAS, Katz’s bADL’s, IADL’s, EURODEM, Risk Factors Questionnaire.	Primary school, High school or higher. Single, married or living as a couple, divorced, or separated, or widowed	Vascular disease (angina, myocardial infarct, and/or stroke), diabetes, hypertension, health status, depression, cognitive status, vascular risk factors (smoking, statin use, body mass index, and alcohol intake)	1,828	In men, but not in women, risk of VaD was higher among individuals with anxiety	No description	Santabárbara et al., 2020
Female	≥55.0 (79.8)				2,229			
Male	≥65.0 (no average age description about male)	MMSE, GMS, CAMCOG, DSM-III-R	−	Hypercholesterolemia, hypertension, or smoking, apolipoprotein E4 allele	12,270	There was no difference by sex in the cumulative risk of vascular dementia. The risk for a 65-year-old woman to develop vascular dementia at the age of 95 years was 0.040 compared with 0.041 for a man.	Age, the square term of age, dummy variables for study, smoking, education, self-reported myocardial infarction, and stroke.	Andersen et al., 1999
Female	≥65.0 (no average age description about female)				16,497			
Male	≥60.0 (70.1)	NINDS- AIREN, CASI, IQCODE	Less education (<7 Years), Higher education (≥7 Years)	Age, sex, obesity, hypertension, diabetes, stroke, drinking, smoking, education	845	Sex and a sex-age interaction showed significant effects with respect to probable VaD, but not to probable or possible AD or possible VaD.	No description	Yamada et al., 2008
Female	≥60.0 (72.2)				2,105			
Male	≥65.0 (no average age description about male)	NINDS, Association Internationale pour Ia Recherche et l’Enseignement en Neuroscience’s criteria, CDR scale, DSM-III-R	Education (number of years of schooling)	Age, sex, education in years, apolipoprotein E4 allele, hypertension, high cholesterol, diabetes, Prevalence of cardiovascular and cerebrovascular factors, obesity, stroke, CABG, myocardial infarction	1,322	Vascular factors increase risks for AD and VaD differentially by sex.	Adjusted for all covariates listed in the medical risk factors list	Steinberg et al., 2014
Female	≥65.0 (74.0)				1,801			
Male	≥65.0 (no average age description about male)	MMSE, MRI-based evidence of lacunar state or ischemic WMLs, ADL, IADL, HDRS, Stroop T	Education (years)	Sex, hypertension, diabetes, hypercholesterolemia, coronary artery disease, tobacco smoking, atrial fibrillation, neurologic signs, family history, history of depression.	156	Moderate mocha coffee consumption was associated with higher cognition and mood status in non-demented elderly subjects with VCI. No description regarding relation of sex.	No description	Fisicaro et al., 2021
Female	≥65.0 (no average age description about female)				144			
Male	16–102 (no average age description about male and/or female)	The Cross-Cultural Cognitive Examination and National Institute of Neurological and Communicative Disorders and Stroke and the Alzheimer’s Disease and Related Disorders Association Alzheimer’s criteria	−	Sex, type 2 diabetes, lifestyle, cigarette smoking, and obesity	2,310,330 individuals, and 102,174 dementia case patients (no separate male and female number description)	Women with diabetes had a 19% greater risk for the development of vascular dementia than men.	Age, race, systolic blood pressure, self-report diabetes; total cholesterol, hypertension, alcohol.	Chatterjee et al., 2016
Female								
Male		STROBE, ARIC, CARDIA, CHS, FOS, NOMAS.	Education (≤8th grade, grades 9–11, completed high school, some college but no degree, ≥College graduate)	Age, race, education, alcoholic, cigarette smoking, any physical activity, BMI, waist circumference, history of arial fibrillation, LDL cholesterol, antihypertensive medication,	11 775	The results of this cohort study suggest that women may have greater cognitive reserve but faster cognitive decline than men, which could contribute to sex differences in late-life dementia.	No description	Levine et al., 2021
Female					14 313			

*MMSE, Mini-Mental Status Examination; GMS-B, Geriatric Mental State B; AGECAT, Automated Geriatric Examination for Computer Assisted Taxonomy; HAS, History and Aetiology Schedule; bADL’s, Katz’s Index for basic activities of daily living; IADL’s, the Lawton and Brody scale for instrumental activities of Daily Living Scale; EURODEM, European Studies of Dementia; GMS, Geriatric Mental State Examination; CAMCOG, Cambridge Examination of Mental Disorders Cognitive Test; CASI, Cognitive Abilities Screening Instrument; IQCODE, Informant Questionnaire on Cognitive Decline in the Elderly; CABG, Coronary Artery Bypass Graft surgery; NINDS, National Institute of Neurological Disorders and Stroke; CDR, Clinical Dementia Rating; WMLs, White Matter Lesions; ADL, Activities of Daily Living; IADL, Instrumental Activities of Daily Living; HDRS, Hamilton Depression Rating Scale; STROBE, Strengthening the Reporting of Observational Studies in Epidemiology; ARIC, Atherosclerosis Risk in Communities Study; CARDIA, Coronary Artery Risk Development in Young Adults Study, CHS, Cardiovascular Health Study; FOS, Framingham Offspring Study; NOMAS, Northern Manhattan Study.*

## General Causes and Risks for VaD

Generally, the common causes of VaD are environmental and pathophysiological that affecting the brain vessels, leading to a reduction of supplies like nutrition and oxygen to the brain (Kalaria, 2016; Killin et al., 2016; Caruso et al., 2019). Therefore, some of the risk factors for VaD coexist with the increased risk of stroke, heart disease, aging, atherosclerosis, high cholesterol, high blood pressure, diabetes, genetics, smoking, obesity, and atrial fibrillation. VaD progresses over time and targets cognitive abilities in the brain (Kalaria et al., 2016). This actively illustrates some of the neurological and behavioral changes that patients with VaD may experience such as memory impairment, loss of executive function (like decreased ability to plan), reasoning and organized thoughts and behaviors. Several regulatory mechanisms and cellular signaling are thought to play a role in VCI and VaD, including oxidative stress, neuroinflammation, endothelial dysfunction, hypoperfusion, blood-brain barrier (BBB) disruption, cortical hyperexcitability, and neurotransmitter imbalance (Vinciguerra et al., 2020). In addition, serological markers that can help in the diagnosis of VaD have been discovered in several investigations. Pro-inflammatory metabolites (such NO-related compounds), cytokines (including IL-1, TNF-α, IFN-γ, IL-4, IL-5, IL-8, G-CSF, and MIP-1b), and endothelial dysfunction indicators (like homocysteine) have all been reported to be elevated in VaD patients (Schmitz et al., 2015).

Moreover, common causes and risk factors of VaD change from one sex to another. For example, males have appeared to be slightly at a higher risk of getting VaD than females at a younger age, but women over the age of 85–90 have been shown to have a higher occurrence (Lucca et al., 2020). Therefore, females have high-risk factors that may be associated with preeclampsia, menopause and poorly timed hormone replacement therapy. Additionally, females have a higher risk of VaD with the presence of diabetes, obesity, and hypertension more than males (Gannon et al., 2019). On the other hand, males have a higher risk with the presence of hyperlipidemia, myocardial infarction, and heart disease like stroke, and heart attack (Skoog, 1994).

## Sex-Specific Differences in VaD

Increasing evidence suggests that sex factors may play an important role in the pathogenesis of diseases, including cardiovascular disease and dementia. Sex differences in prevalence also depend on dementia subtypes, such as AD, VaD, Lewy body dementia (LBD), and Parkinson’s dementia (PD) (Kim et al., 2018). Therefore, studies are needed to investigate sex-specific differences, which can help understand the pathophysiology of dementia and identify potential therapeutic targets for both sexes. In response to variables that promote either positive or negative neuroplasticity, an individual’s overall cognitive state might change over time. The physiological ability of the brain to establish and strengthen dendritic connections, cause good morphological changes, and boost cognitive reserve is referred to as positive neuroplasticity. Negative neuroplasticity refers to the same physiological ability of the brain to atrophy and weaken dendritic connections, produce detrimental morphological changes, and decrease cognitive reserve. Many factors promote positive or negative neuroplasticity including sex. Though women showing a greater risk of AD at later ages (Lin and Doraiswamy, 2014), it has been suggested that diagnosis of mild cognitive impairment (MCI) may be delayed in females, which may be responsible for the possible increased risk for MCI seen in males. Specifically, despite identical levels of neurodegeneration, females perform better in verbal memory than males (Sundermann et al., 2016a, b). This study shows that women have a cognitive reserve in this area, delaying the onset of abnormalities until higher levels of disease are present. Although the correlations are varied and multifaceted, there are sex differences in dementia. A study done in rats provides another example, showing that sex variations in neuroplasticity are the response of hippocampal neurogenesis to prolonged estradiol therapy (Barker and Galea, 2008).

With the prevalence of cardiovascular disease in recent years, the risk of VaD is also on the rise. Besides just simply having any form of cardiovascular disease, other factors can help contribute to developing VaD in some indirect ways. Sex can influence the occurrence of VaD, and there are trends that VaD follows between the sexes. Some studies have shown a difference in how the prevalence of VaD is altered by a person’s biological sex (Andersen et al., 1999; Podcasy and Epperson, 2016). The occurrence of dementia and the reasons for differences between the different sexes are complex. Like all dementia diagnoses, the symptoms can vary and are often caused by complex factors. There may not be a particular reason but a culmination of multiple factors at a personal level. For example, a woman experiencing menopause could also be experiencing symptoms of other health conditions that accumulate and lead to a higher risk of VaD (Robusto-Leitao and Ferreira, 2006; Yamada et al., 2008). Her risk is possibly higher than a male of similar age who only has hypertension. Additionally, another factor to consider besides age category risks is that females live 6+ years longer than males on average, so their occurrence of VaD is higher based on longevity (Robusto-Leitao and Ferreira, 2006). These factors only consider physical factors, there may be many more, including educational level and socioeconomic status, etc. These additional factors can be attributed to a more social environment aspect of VaD. For example, the opportunities available for women vary in the sense of a generational gap. Females were placed into a social role of caretaking the household and had less physically demanding work roles than men (Hörder et al., 2018). Therefore, the risk of VaD could increase for women. Also, the occurrence of smoking has changed with time, it was less socially acceptable for women to smoke tobacco and other products but in recent years the number of female smokers has increased. These factors lead to a higher vascular risk and could be directly related to higher dementia cases in women in the long run (Zhong et al., 2015). Lastly, the sex differences noted in dementia are not really prominent until after the age of 80, and that is only consistent in studies done in the United States (Podcasy and Epperson, 2016; Levine et al., 2021). Other studies in Europe show a sex difference in VaD early on, indicating that the occurrence of VaD can be influenced by external factors (Hasselgren et al., 2020). It is highly likely that each VaD diagnosis is unique and is a combination of both social and physical factors that affect an individual’s neurobiology (Table 2).

**TABLE 2:** General risk factors of VaD.

**Vascular dementia common risk factors**	
Genetics	Genetics can cause VaD depending on the presence of certain gene variants. This includes ApoE alleles and TDP43.
Obesity	Obesity leads to VaD due to higher blood pressure. Over time this can cause ventricular enlargement and atherosclerosis.
Hyperlipidemia	Hyperlipidemia blocks blood flow to the brain which overtime increases the chances of developing VaD.
Diabetes	Type II diabetes is highly associated with VaD because of abnormal blood flow to the brain. Type I is associated but not as highly as Type II.
Hypertension	Hypertension increases the risk of stroke which is a huge risk factor for VaD.
Stroke	Stroke impedes blood flow to areas of the brain. The oxygen deprivation destroys the brain tissue.
Atherosclerosis	Atherosclerosis prevents blood from reaching the brain fully. This deprives the tissue of the oxygen and nutrients.
Atrial Fibrillation	Atrial fibrillation carries and increased risk of stroke which is directly linked to vascular dementia.
Aging	Aging can increase the chance of VaD because there is a higher risk of atherosclerosis, heart attack and strokes.
Smoking	Smoking increases the risk of stroke but additionally toxins in cigarette smoke cause oxidative stress and inflammation, both associated with VaD.

*All these factors can overlap and when combined the risk of developing VaD increases.*

### Pre-existing Health Conditions/Family History Risk Factors for VaD

Perhaps one of the most common risk factors with any disease or condition is family history. A person’s family history can indicate common trends and probabilities about a person’s health, indicating what kind of lifestyle is best suited to everyone’s needs. Indirectly a family history of cardiovascular disease can put a person, regardless of their sex, at a disadvantage for developing VaD because CVD is a hereditary risk factor that becomes a more significant risk factor for VaD (Treiber et al., 2008; Vijayan and Reddy, 2016). In terms of family history there are not many direct links of inherited conditions that can contribute directly to VaD. The only direct familial link to VaD occurs with gene variants and specific gene mutations (Lin et al., 2019). Additionally, the pre-existing health conditions that can contribute to VaD are smoking, obesity, hyperlipidemia, and old age (Tariq and Barber, 2018). Moreover, the factors mentioned are not specific to one sex but have been sorted by the sex in which the factor is more dominant (Podcasy and Epperson, 2016).

#### Risk Factors for Males and Females

The cardiovascular risk for VaD in women is less than that of men, but the prevalence varies (Steinberg et al., 2014). In the United States, men have a greater risk of having a heart attack and are twice more likely to have a heart attack than a women, yet the risk for CVD/heart disease is similar (Maas and Appelman, 2010). For smoking, worldwide, the trend has been consistent with men in developed countries smoking less than men in developing countries (Pampel, 2006). The use of tobacco products restricted to just the United States indicates that men smoke slightly more than women.

Researchers discovered a significant difference in age and education among the four groups of subjects based on their daily mocha coffee consumption. However, the General Regression Model revealed that the associations between coffee consumption and cognition and mood were independent of socio-demographic variables (Fisicaro et al., 2021). In terms of the sex-related effect, an earlier prospective study (Shinoda et al., 2015) of 455 participants (314 men) found that male drinkers had a lower incidence of small vessel disease than male non-drinkers and occasional drinkers, while female drinkers had a lower incidence of white matter lesions (WMLs) than female non-drinkers or occasional drinkers.

Obesity as a risk factor is prevalent in women, and overall globally, women tend to be more obese than men, but not in highly developed countries. The risk for smoking is also directly inverted for women. Women in developed countries, smoking more than currently developing countries. However, the gap within the United States of men and women smoking varies only by about 3% (Mackay and Amos, 2003). Lastly, in terms of hyperlipidemia (Davidson et al., 2002) and anxiety (Santabárbara et al., 2020) condition, even though men have a higher risk for VaD, and it is thought of as a man’s disease, women are still at risk for high cholesterol, and the risk increases in women after menopause or early menopause. It is believed that younger women (or just generally those who have not reached menopause yet) are protected from more cardiovascular events than men due to the presence of high estrogen (El Khoudary et al., 2020).

#### Overlapping Risk Factors

As previously mentioned, there is no specific family history indicative of a high probability of developing VaD, besides CVD. However, there is not a significant difference between males and females in this area. The percentage of lifestyle-influenced risk factors that contribute to VaD in the United States is mentioned in Table 3 (Whitmer et al., 2007; Gannon et al., 2019; Lewis et al., 2021). With these factors, other factors interrelated to family histories like gene variants and gene mutations can help contribute to VaD.

**TABLE 3:** Percentage of lifestyle influenced risk factors that contribute to VaD.

	**Males**	**Females**
**Obesity**	35%	40.4%
**Hyperlipidemia**	28.5%	8.9%
**Smoking**	16.7%	13.6%

### Gene Variants as a Risk Factor for VaD

Much work has been done on gene variants in AD, and the effects of these genes in VaD have been rendered. The leading players in VaD include a mutation in the Notch Receptor 3 (NOTCH3) gene, which is directly responsible for one form of VaD, and Apolipoprotein E (APOE) variants (Skoog et al., 1998; Thomas et al., 2000; Cho et al., 2021). NOTCH3 is directly linked to cerebral autosomal dominant arteriopathy with subcortical infarcts and leukoencephalopathy also known as CADASIL (Papakonstantinou et al., 2019). CADASIL is considered a very rare disease and has been recorded across all ethnic groups thus far (Kalaria et al., 2004). It is most often caused by a missense mutation but can alternatively be caused by null mutations or homozygous mutations. The symptoms of CADASIL include smaller cerebral vessels which in turn cause strokes, mood disturbances, and of course VaD (Dichgans, 2002). The other gene attributed as a risk factor for VaD is APOE and its variants (Skillbäck et al., 2018; Montagne et al., 2020). APOE has different variants denoted by e2 (APOE2), e3 (APOE3), and e4 (APOE4).

**FIGURE 3:**
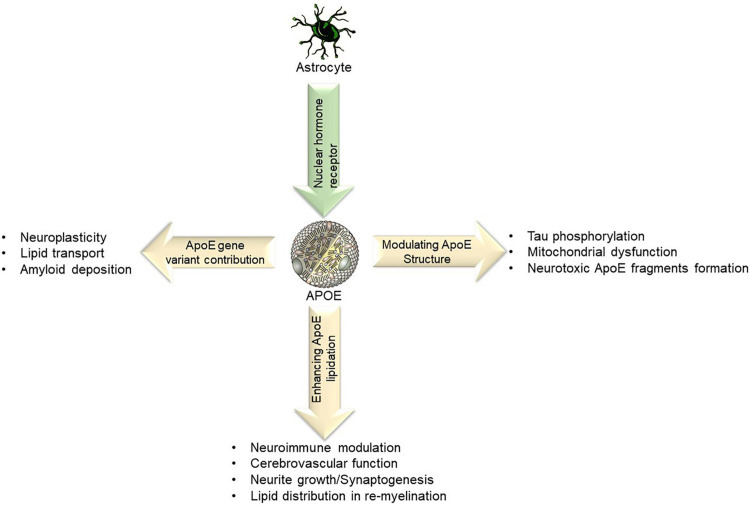
Lipid transport refers to the ability of APOE to bind to LDL receptors and assist with lipid clearance. Certain APOE alleles also interact and affect amyloid deposition, neuroplasticity, neuroimmune modulation and in re-myelination processes.

Astrocytes predominantly express APOE variants (APOE4) in the brain, cell types that are now recognized to play critical roles in largely lipid distribution, cerebrovascular function amyloid deposition, Tau phosphorylation, mitochondrial dysfunction (Fernandez et al., 2019), as depicted in Figure 3. Most of the research surrounding APOE has been centered around AD, however, the same relationships established between APOE and AD apply to VaD but in a lesser magnitude (Alam et al., 2014). For example, APOE4 has been the most prominent contributor to AD, and when two copies of the APOE4 allele are present the risk for AD increases 15-fold. This increase is directly analogous to VaD because the same APOE e4 variant is the same variant that influences the risk for VaD. However, the effect is weaker and does not increase the risk as dramatically as 15-fold (Rohn, 2014).

#### Risk Factors for Males and Females

Males have a decreased risk of VaD in carrying an APOE4 variant even if the male is homozygous, the link between APOE and AD/VaD is well established with females and not males (Rasmussen et al., 2018). This is such an important factor that even most studies involving tracing APOE to dementia are done with female mice (Adeosun et al., 2019). Most literature also supports that APOE2 and APOE3 are neutral factors in males developing VaD (Folin et al., 2004; Bell et al., 2012). In terms of the mutation for CADASIL, men have equal risk, but the symptoms of CADASIL affect men differently (Ruitenberg et al., 2001; Singhal et al., 2004). Men have more strokes on average than women at a younger age and retain cognitive ability better than women after a certain age (typically when comparing the sexes before menopause). APOE4 carries the highest risk for women developing dementia, especially for women who are homozygotes and carry two e4 alleles (Molero et al., 2001; Davidson et al., 2006). It is estimated that the risk increases 10-fold for VaD and an earlier onset of dementia if a person is homozygous. APOE2 is the least common allele but provides a protective effect against dementia and cognitive decline in women (Volgman et al., 2019). CADASIL affects women mainly by increasing the occurrence of frequent migraines accompanied by vision impairment (Rufa et al., 2004; Guey et al., 2016). This is most prevalent until the age of menopause and then cognitive impairment becomes more evident. The occurrence of psychiatric symptoms and migraines increase after menopause, but women, however. have a decreased rate of strokes in comparison to men (Bushnell et al., 2014).

#### Overlapping in Gene Variants

Overall, both males and females have an equivalent risk of having CADASIL. The symptoms do vary, and that aspect is dictated by the age of the male or female. There also was no inconsistency between sex and the type of mutations that caused CADASIL (Mizuno et al., 2020). When it comes to APOE as a contributor to VaD, however, there is a significant difference between males and females (Molero et al., 2001). Females have a higher risk than males when it comes to having variant APOE4 and females had a decreased risk when they carried APOE2. Males had a generally neutral experience when carrying alleles of APOE2 and APOE3. Lastly, APOE4 was not shown to be as much of an indicator in males for VaD as much as females (Molero et al., 2001; Li et al., 2017).

### Aging as a Risk Factor

Aging is one of the leading and highest risk factors of VaD because of the association of adiponectin (Song et al., 2014). Adiponectin is a protein hormone secreted by adipose tissue (fat cells). In aging, adiponectin plasma levels decrease, leading to an increased risk of CVD and diabetes, considered a risk factor of VaD (Song et al., 2014; Chatterjee et al., 2016; Chen et al., 2019). Additionally, adiponectin plays a massive role in cognitive dysfunction by controlling the insulin signal transduction in the brain (Rizzo et al., 2020). An epidemiology study implies that there is an exponential increase in dementia after the age of 65 by double around every 5 years. However, for participants aged 90 and older, dementia doubles every 5 years for females but not males (Corrada et al., 2010).

Vascular dementia and multi-infarct dementia are most common in males but have a higher gravity of impact in females. The study concluded that stroke prevalence, whether ischemic or hemorrhagic, was 44% greater in males than in females worldwide. Also, males experience their first strokes at a younger age than females (68.6 vs. 72.9 years) (Persky et al., 2010; Girijala et al., 2017). Although males’ first stroke experience might be at a younger age than females, females have a higher risk of lifetime stroke because of their longer life expectancy, which also increases the risk of stroke with age (Reeves et al., 2008).

### Hormone Fluctuations as a Risk Factor

Studies have been done to investigate the controversy between correlating estrogen changes to cognitive impairment, which causes VaD (Ali et al., 2018). The reason for this occurrence is the sudden downfall of estrogen in women during menopause; this happens to be one of the significant risk factors and the root prevalence of VaD in postmenopausal females. Furthermore, a few studies concluded that estrogen deficiency in postmenopausal women is indeed one of the risk factors, causing cognitive deficit that can lead to VaD (Robusto-Leitao and Ferreira, 2006). The four-core genotype (FCG) model is used to separate the contributions of sex hormones and sex chromosome complement (Bushnell et al., 2018). The sex determining SRY gene is relocated from the Y chromosome to an autosome in this scenario, allowing XX males (XXM) and XY females (XYF) to be produced. Because the created XXM have a hormonal status like that of XY males (and vice versa for XYF and XX females), a comparison between the two provides for a better understanding of chromosome vs. hormone impacts. In addition, sex chromosomes have received little consideration in connection to the pathogenesis of dementia (Rocca et al., 2014). Women have two copies of chromosome X, one from their mother and one from their father. The androgen receptor and many proteins involved in mitochondrial function, adipose tissue distribution, apoptosis, and sensitivity to hypoxia are among the approximately 1,600 genes (about 155 million base pairs) on the X-chromosome (Wise et al., 2013). In female cells, most of the genes expressed on one of the two X-chromosomes are inactivated to avoid a genetic overload (Brown et al., 1991). As a result, X-chromosome inactivation patterns may provide a new viewpoint on the concept of laterality of brain activities in women vs. men.

#### Male vs. Female Hormonal Risk/Benefits

Females seem to be more susceptible overall to dementia than males. The association between sex hormones and VaD is still somewhat unknown in the scientific/medical field. While there is extended gratitude to consider sex as one of the factors of VaD, there are various factors that might distinctively impact females, such as pregnancy and reproductive history.

A study was focused on a comparison between serum sex hormone levels among VaD patients (males and females) and normal (controls) individuals to bridging the gap between sex hormones level and cognitive and neuropsychiatric manifestations of VaD (Xing et al., 2013). The results of the study showed that the testosterone and sex hormone-binding globulin (SHBG) levels were lower in male VaD patients, and the estradiol levels were found to be higher in female VaD patients in comparison to the controls. The study concluded that there were no correlations between hormone levels and neuropsychiatric symptoms in male VaD patients, whereas total estradiol (TE2) and testosterone (TT) levels were positively correlated in female VaD patients. Estrogens influence the function and pathophysiology of cerebral circulation. Estrogen decreases cerebral vascular tone and increases cerebral blood flow by enhancing endothelial-derived nitric oxide and prostacyclin pathways (Krause et al., 2006). In addition, however, blood vessels produce inflammatory factors that could contribute to VaD pathology. One study suggests that estrogens and their receptors may regulate the neuroinflammatory response, and in females, circulating estrogens may play a protective, anti-inflammatory role (Villa et al., 2016). Thus, estrogen preserves vascular function and neuroinflammatory response could be directly relevant to VaD.

Another study on animal models suggests that young adult male mice had worse pathological and functional outcomes following cerebral ischemia than females, which is consistent with clinical studies suggesting that high-androgen levels enhance stroke risk in younger populations (Abi-Ghanem et al., 2020). These sex differences could be due to sex hormones or sex chromosomal complement.

#### Hormone Therapy Risks/Benefits for VaD

Hormone replacement therapy (HRT) can be used for a variety of reasons, even to deal with symptoms of the menopause. As previously mentioned, women have protection against forms of dementia due to their high estrogen levels. Once menopause is reached, however, those estrogen levels deplete. In these cases, women can have added protection against VaD despite their initial lower estrogen. A few studies finding a correlation have analyzed the relationship between HRT, AD and VaD specifically. One study achieved that estradiol-based HRT was associated with a reduced risk of death both from VaD and AD, but the risk reduction was larger and appeared sooner in VaD than AD (Mikkola et al., 2017). In a few studies, the use of estrogen post-menopause decreased the user’s risk for dementia and improved cognitive decline (Yaffe et al., 1998). Another study suggested that the prior use of estrogen was more effective in combating dementia and cognitive decline as opposed to using HRT during post-menopause (Henderson, 2008). Most types of HRT increase the risk of breast, ovarian and, womb cancer (D’Alonzo et al., 2019). But the risk is higher for those using combined HRT, which uses both estrogen and progestogen. The risk of cancer due to HRT can also vary from person to person. Things such as what age you are when you first start taking HRT, other medicines you may be taking, and your general health can impact the risk. People who begin HRT before or soon after menopause may have a bigger risk than those who start HRT later (Beral et al., 2011). In one such study, HRT was analyzed in postmenopausal women and the results indicated that HRT contributed to vascular risk factors including stroke and could not sufficiently demonstrate benefits for postmenopausal women regarding dementia (Hogervorst et al., 2000). The results are clearly divided for the use of HRT, but the main conclusion most studies come to is that HRT can be beneficial if used within a proper time frame to protect postmenopausal women from dementia forms.

### Environmental Factors as a Risk Factor

The physical risk factors of VaD are only one aspect of the cause. VaD can arise due to many factors but one class of factors that are often overlooked are the environmental factors which often can dictate some of the other more tangible, direct factors (Killin et al., 2016). Some environmental risk factors include air quality elements, toxic heavy metals, trace elements, known occupational hazards, electrical/magnetic fields, and a few others (Killin et al., 2016; Bellou et al., 2017; Wang et al., 2019). These different environmental factors have been shown to have strong effects in propagating dementia (Table 4).

**TABLE 4:** Shows the various types of environmental factors and their uses.

**Environmental factor**	**Factor**	**How it is produced/or used**
Air Quality	Nitrous Oxides	Combustion, especially in areas of high motor vehicle use
	Ozone	UV Radiation reactions with oxygen and sometimes nitrogen oxides
Toxic Metals	Arsenic	Industrial use in mining and ore smelting
	Aluminum	Industrial mining and smelting
Trace Elements	Silicon/Silica	Industrial silicon and silica working introduces fine particles into the air
	Selenium	mining/oil refining
Work Hazards	Pesticides/Herbicides/insecticides/fertilizers	General farming and crop production, can be endocrine disrupting
	Solvents/degreasing agents	Industrial grade chemical for various work trades

The factors listed in the table are the highest environmental factors that have a link to VaD, and they all are produced in different ways, some of which cannot be simply avoided. These factors can trickle down, and most likely do not directly affect the development of VaD but can exacerbate other existing risk factors. For example, the use of herbicides, pesticides, insecticides, and fertilizers are commonly used in farming practices all over the world, many of these are known endocrine disruptors that increased the risk of developing dementia (Kamel and Hoppin, 2004).

Other less obvious environmental factors can include weather patterns that govern a certain area as well as Vitamin D level deficiencies from lack of sunlight exposure (Sommer et al., 2017). The area in which a person lives can also influence the risk of VaD. For example, one study done in China indicated that in areas with less particulate that the people in those areas had a reduced occurrence of cerebrovascular events (Cai et al., 2018). Reduced risk of cerebrovascular events decreases the risk of VaD because if harmful vascular events do not occur, there is no disruption to brain tissue. Another study showed that with extremely low vitamin D levels patients were more likely to develop VaD and more likely to develop vascular events that perpetuated the occurrence of VaD (Chai et al., 2019). Another study indicated that warmer and wetter weather conditions provided for a better foundation for stroke patients to recover fully (Chen et al., 2013). This is important because stroke is one of the major contributors to VaD (Vijayan and Reddy, 2016). By making a full recovery from a stroke the risk of VaD is mitigated. Lastly, in one study air pollution and noise were linked to a progression of dementia in London, England (Carey et al., 2018). These results were more consistent with an AD diagnosis but there was a fair amount of VaD cases present as well.

## Accessible Treatments for VaD

### Pharmaceutical Treatments

Many drug categories have been used as a treatment for VaD patients. Some of these drug categories are vasodilators, calcium channel blockers, antiplatelet, etc. (Romàn, 2000; Chabriat and Bousser, 2006; Nimmrich and Eckert, 2013; Table 5). One of the most effective and standard treatment are cholinesterase inhibitors (Bullock, 2004). Cholinesterase inhibitors work by inhibiting acetylcholinesterase, which is accountable for clearing acetylcholine, a neurotransmitter responsible for muscle contractions, blood vessel dilation, and regulating heart rate. With the cholinesterase inhibited, the acetylcholine concentrations rise and lead to better communication between the nerve cells in the brain. An artificial increase in acetylcholine levels by physostigmine, an acetylcholinesterase inhibitor that increases the extracellular acetylcholine levels, impairs memory consolidation and rescue in rodent and human subjects (Haam and Yakel, 2017). Vasodilation drugs work mainly in preventing constriction of the blood vessels anywhere in the body and allowing greater blood flow. However, calcium channel blockers prevent calcium from entering the cells in the heart, vascular smooth muscle, and pancreas by lowering the blood pressure. Aspirin is a popular antiplatelet drug that works by blocking the movement of the cyclooxygenase chemical (COX) via the prostaglandin synthesis pathway (PGH2) (Warner et al., 2011; Ornelas et al., 2017).

**TABLE 5:** Pharmacological treatment for VaD and their effects.

**Drug classes**	**Drugs**	**Effects**
Cholinesterase inhibitors	Donepezil, Galantamine, Rivastigmine	Safe and effective in reducing the progression of VaD in addressing the behavioral problems.
Vasodilators	Niacin (nicotinic acid), Cyclandelate, Papaverine, Isoxsuprine, Cinnarizine, Buflomedil, Naftidrofuryl, Ergoloid mesylates, Acetazolamide	Have shown to be less effective in treating VaD compared to cortical dementias like Alzheimer’s.
Calcium channel blockers	Nimodipine	Moderately effective for short term in treating cerebrovascular disease and other types of dementia.
N-methyl-D-aspartate (NMDA) antagonists	Nicardipine	Improvement of cognitive deterioration
NMDA antagonists	Memantine	Improve cognition consistently across different cognitive scales, with at least no deterioration in global functioning and behavior.
Nootropic agents	Piracetam, Nicergoline, Oxiracetam Citicoline, Pentoxifylline	have beneficial effects for patients with dementia in prolonging their survival.
Antiplatelet agents	Aspirin, Triflusal, Ginkgo biloba	Reduce/prevent the occurrence of stroke.

The drugs that are mentioned in the table above are only the pharmaceutical treatments for VaD (Olivares et al., 2012; Wu et al., 2015). There have been a few studies indicating alternative treatments that have had success. The alternative and homeopathic treatments mostly treat the symptoms of the VaD but occasionally can treat the vascular underlying conditions (McCarney et al., 2003). A study done in 2018 concluded that acupuncture could in fact help to treat VaD and it generally worked by boosting the metabolism of glucose and oxygen, contributing to anti-oxidative stress reactions, and acted as an anti-apoptotic agent (Zhu et al., 2018). In another study, aromatherapy essential oils (rotated lemon, rosemary, lavender, and orange oils) were given to dementia patients topically throughout the day for a period of 28 days (Jimbo et al., 2009). After 28 days the aromatherapy was shown to have improved the patient’s personal orientation and cognition. This improvement in the patients could be applied specifically to VaD and the treatment proved to be an efficacious non-pharmacological treatment. Music therapy was analyzed for its effect on dementia patients indicated that having at least 5 music therapy sessions a day reduced depression and generally improved the quality of life of dementia patients (van der Steen et al., 2018). The effects of music therapy on actual cognitive function were unknown, however. Lastly, art therapy was another means of homeopathic therapy, functioned similarly to music therapy in that it improved the patient’s quality of life (Deshmukh et al., 2018). The actual cognitive function could not be measured but the therapy improved neuropsychiatric symptoms of dementia patients. While homeopathic remedies are a reasonable alternative, they tend to focus on the symptoms rather than the triggers. The causes of risk factors might be discussed in order to completely eliminate the risk of VaD. Thus far, there is no drug available related to sex/gender and therefore, the development of pharmaceutical treatment for VaD has become an essential but unmet need.

### Elimination of Risk Factors

Eliminating a risk factor should zero in on a reduction of the major causes of VaD such as stroke, and CVD, with consideration regarding controlling of the other risk factors. One of the biggest risk factors for VaD is arterial hypertension (Paglieri et al., 2004). Arterial hypertension is the main cause of 50% of strokes regardless of the pathogenic mechanism involved. Also, lower blood pressure decreases the risk of VaD by 55% (Sierra, 2020). Indeed, the primary prevention focus is to decrease the prevalence of VaD by early and optimum treatment for stroke and CVD by targeting high-risk groups such as the elderly, hypertensive patients, diabetes patients, smokers, past transient ischemic attack or stroke survivors, hypercholesterolemia patients, and atrial fibrillation patients (Vijayan and Reddy, 2016).

The primary prevention aims to minimize dementia in the population by advising lifestyle changes such as the control of diabetes and hypertension (Eggink et al., 2019). The secondary prevention methods aim to mark stroke management and the prevention of recurrent stroke (Lip and Kalra, 2010). This has shown a 50% decrease in the risk of dementia in those with previous stroke experiences and a 16% decrease in those without stroke experiences in patients without cognitive impairment (Lo Coco et al., 2016).

The specific ways that dementia patients or dementia patient caregivers are prescribed to help reduce symptoms include lifestyle changes. These lifestyle changes are aimed at improving the vascular health of the body to reduce any kind of negative events like stroke or heart attack. The methods include diet changes, incorporating exercise either vigorous or more passive, cognitive therapy, and management of vascular issues like hypertension through medication. A research study published in 2016 demonstrated the effectiveness of integrating exercise into a stroke rehabilitation environment (Winstein et al., 2016). Stroke patients who included moderate aerobic activity and environmental enrichment in their treatment plans had higher recovery rates and, in some cases, spontaneous recovery. Another research published in 2018 found that incorporating passive exercise into a dementia patient’s everyday routine resulted in substantial improvements in quality of life and activities of daily living (Zucchella et al., 2018). Most of the patients in this study improved their cognitive and physical functioning. A study done in Finland called the FINGER trial was aimed at improving the outcomes in VaD patients through a total reset of the patient’s lifestyle (Kivipelto et al., 2020). With regular medical advice, intervention (diet, exercise, vascular risk management, lifestyle guidance, intellectual training, social activities, cognitive training, prevent head injury, stop smoking, reduce air pollution), and multi-domain intervention, cognitive function was partially restored. Another method to reduce symptoms of VaD includes cognitive therapy was done in 2019 aimed to test individuals with mild to moderate VaD and to view if there were any cognitive changes (Peng, 2019). There was no net improvement in the cognition of the dementia patients, but of those that had caregivers, the moods of the caregivers were improved and in turn led to a better mood and outlook for the dementia patients. As for medication, several drugs have been prescribed to treat VaD after the risk factors have been addressed. Since zinc deficiencies play a critical role in neurodegenerative diseases, a study published in 2018 found that carnosine and zinc, which are commonly used as a supplement to treat gastric ulcers, may be used as a future remedy for VaD (Kawahara et al., 2018). Lastly, in a study done in 2016, amlodipine, a common hypertension treatment was given to VaD patients (Fares et al., 2016). There was a slight improvement in the symptoms of VaD patients. Even though there is little research on non-traditional therapies, there is still a lot of importance in looking into the options with VaD since there is no standardized care. VaD is very challenging to treat, and this is partially due to a lack of understanding of the full pathophysiology.

## Conclusion

With the rise of VaD cases across the globe, it is easy to see why researchers have taken an interest in its risk factors. An overlooked factor is often sex and gender. In this review, we explored the impact of sex/gender differences on VaD, including how they affect these risk factors: pre-existing health conditions and family history, gene variants, aging, hormone fluctuations, and environmental risk factors. Improvements in the following areas may help to reduce the prevalence of VaD: (1) stroke prevention in hypertensive patients, (2) appropriate treatment to prevent or slow atherosclerosis progression, (3) a better understanding of the relationship between menopause and hypertension control, and (4) efforts to reduce neurological complications associated with cardiac procedures, (5) avoiding poorly timed hormone replacement therapy (6) evading potentially dangerous drugs that impair memory and cognition, and (6) developing therapy targets based on a better understanding of the molecular function of the apolipoprotein E4 genotype in dementia. These all play a significant role in developing VaD and additionally should be monitored to reduce the risk. When it comes to sex and gender there is not a fine line between how VaD presents itself. VaD is a multidimensional illness with many different moving pieces, and every case of VaD has its risk factors that can be attributed to a plethora of lifestyle factors and chance. Future research should be focused on treating VaD by assessing the occurrence of risk factors in sex and a variety of other lifestyle indications to create a more tailored treatment plan. If enough research is done, a strong connection between sex and VaD could be discovered, which may aid in improving current therapies and developing unique sex-based treatment plans. Additionally, more research should also be conducted on how socioeconomic factors impact a patient’s physiology and how that impacts their ability to develop VaD. This could be important due to how social constructs can dictate health based on sex. For example, females up until recently had a more social role of refraining from smoking to maintain a feminine image. In addition, more research should be planned on possible gene therapies and how the occurrence of VaD affects the LGBTQ+ community and changes their physiology. This would be particularly salient in studying the occurrence of all types of dementia and VaD in the transgender population and for individuals that use hormone replacement therapies. Lastly, future trials must examine sex-specific differences to improve our understanding of how CVD affects cognitive impairment in women and men.

## Author Contributions

FA, AP, and YZ drafted the manuscript and figures. ZZ and DZ proofread and revised the manuscript. DZ gave the final proof for this submission. All authors contributed to the article and approved the submitted version.

## Author Disclaimer

The content is solely the responsibility of the authors and does not necessarily represent the official views of the National Institutes of Health.

## Conflict of Interest

The authors declare that the research was conducted in the absence of any commercial or financial relationships that could be construed as a potential conflict of interest.

## Publisher’s Note

All claims expressed in this article are solely those of the authors and do not necessarily represent those of their affiliated organizations, or those of the publisher, the editors and the reviewers. Any product that may be evaluated in this article, or claim that may be made by its manufacturer, is not guaranteed or endorsed by the publisher.
